# Bleeding frequency during physiotherapy in thrombocytopenic patients undergoing hematopoietic stem cell transplantation

**DOI:** 10.1371/journal.pone.0255413

**Published:** 2021-07-30

**Authors:** Erick Alvaro Grencheski, Margarete Noriko Kochi, Flávia Vanessa Aurea Politi, Tabata Maruyama dos Santos, Christina May Moran de Brito, Wellington Pereira Yamaguti, Renato Fraga Righetti

**Affiliations:** 1 Hospital Sírio-Libanês, Rehabilitation Service, São Paulo, Brazil; 2 Instituto do Câncer de São Paulo (ICESP), São Paulo, Brazil; The University of Texas MD Anderson Cancer Center, UNITED STATES

## Abstract

**Background:**

During hematopoietic stem cell transplantation (HSCT) the patients perform activities of low and moderate intensity because have reduced hematological lineages, leaving them susceptible to hemorrhagic events. The objective of this study was to describe the frequency of bleeding events, severity, and possible association with physical exercise in thrombocytopenic patients.

**Methods:**

A retrospective study with seventy-seven HSCT patients hospitalised, that had a platelet count ≤ 50,000 /μL and received physical exercise during physiotherapy intervention.

**Results:**

Regarding bleeding events, only six were related to physical exercise, and bleeding events occurred more frequently at platelet levels ≤ 10,000 /μL. The most frequent bleeding event was epistaxis, considered of low severity, and with the moderate possibility of being related to physical exercise; followed by extremity hematoma, considered of medium severity and highly related to physical exercise. In this study, there was no occurrence of bleeding events considered of high severity.

**Conclusion:**

Bleeding frequency in supervised physical exercise during physiotherapy in adults with thrombocytopenia undergoing HSCT is minor and relatively rare but occurs more frequently in patients with platelet count ≤10,000 /μL. These results encourage the maintenance of physical activity in this population who is at high risk of developing immobility-related complications.

## Introduction

Hematological neoplasms comprise a wide category of alterations that affect the functioning of the bone marrow and lymphoid organs, compromising the production and function of hematopoietic cells [1, 2]. In 2018, the World Health Organization (WHO) estimated 509,590 cases of Non-Hodgkin’s Lymphoma and 437,033 cases of Leukemia [3]. In Brazil, epidemiological data of the National Cancer Institute (INCA) estimated 5,940 new cases of leukemia for the 2018–2019 biennium, the most prevalent hematological neoplasm, followed by Non-Hodgkin and Hodgkin’s lymphomas, respectively [1].

The treatment of hematological malignancies involves intense medical regimens, and in some cases, Hematopoietic Stem Cell Transplantation (HSCT), resulting in extended periods of hospitalization, high risk of infection and re-hospitalizations, interfering with biological, psychological and social aspects of patients and family members [2, 4]. During the pre-and post-HSCT period, the recipients are susceptible to the toxicities of conditioning with myeloablative chemotherapy, radiotherapy, and complications resulting from HSCT, which have deleterious effects on the cardiorespiratory and musculoskeletal system. Also, the time of prolonged inactivity causes the decline of physical and functional capacity, affecting the quality of life of the patient [5, 6]. Additionally, these patients develop severe thrombocytopenia, increasing their predisposition to bleeding [7, 8].

For a long time, absolute rest has been indicated for thrombocytopenic patients; however, studies indicate a strong correlation between immobility and a vicious cycle of physical dysfunction, which results in a rapid and irreversible loss of function, increased morbidity, and mortality [9, 10]. In this context, studies have demonstrated the positive effect of early physiotherapy interventions in HSCT recipients [11, 12] showing that physical exercise can reduce the decline in physical performance, cytopenia, and hospital stay [13, 14]. However, safety levels of platelet counts and erythrocytes for physical exercise are not fully established in the literature [15]. Current publications propose that patients with platelet counts between 50,000–30,000 /μL perform exercises of moderate-intensity and active range of motion; 30,000–20,000 /μL walking, non-strenuous exercises, and fall prevention; and <20,000 /μL activities of daily living with caution for the risk of falls [16].

The literature is scarce about the safety and complications of physical exercise in patients with platelet counts less than or equal to 50,000 /μL. Thereby, studies that evaluate the frequency of bleeding events, their severity, and possible association with physical exercise in thrombocytopenic patients before and after HSCT, during the hospitalization period are necessary. This study aimed to describe the bleeding frequency, its severity, and its association with the physical exercise during physiotherapy intervention of thrombocytopenic HSCT recipients hospitalized in a Bone Marrow Transplant (BMT) unit.

## Materials and methods

### Ethical aspects

The study follows the ethical precepts established by the National Health Council (NHC) by number 466/12 and was approved by the Institutional Research Ethical Committee of the Hospital Sírio-Libanês (number 2,116,361).

### Characterization of the study

It is characterized as a retrospective study using a sample of hospitalised patients in the BMT unit in a private hospital, São Paulo, Brazil, who received physical exercise during physiotherapy intervention between January 2011 and December 2015. The study included patients aged ≥ 18 years, who had undergone HSCT, that had a platelet count ≤50,000 /μL. They were observed from the introduction of the HSCT conditioning until 30 days after the transplant. Patients were divided into five groups according to the degree of thrombocytopenia (≤10,000; 10,001–20,000; 20,001–30,000; 30,001–40,000; 40,001–50,000 /μL). We excluded patients who had an incomplete or unavailable medical history and/or prior coagulopathy.

### Data collection

For data collection, an instrument form was developed by the authors based on information from the literature and from the professional experience of the researchers and multidisciplinary team. The information contained in the electronic medical records of each patient was accessed to fill out the form.

The instrument form presented the following items:

- Patient’s medical chart number.
- Age, gender, weight, height, and ethnicity.
- Baseline disease that led to the indication for HSCT and the type of HSCT.
- Laboratory information including daily platelet count.
- Medical information including a description of events, including bleeding episodes.
- Description of hemorrhagic complications (type, severity, and complications) that occurred during the conditioning period and 30 days after HSCT.

The instrument forms were filled by the researchers daily on information on medical, physiotherapeutic, and nursing team records. Hematological complications were considered to be related to physiotherapy if their relationship was observed and registered. Every occurrence of a bleeding episode was computed; however, if the same event of bleeding in the same patient was documented in multiple days in the medical records, it was considered only as one event.

In this study, any physical exercise supervised by the physiotherapist during hospitalization at the BMT unit was considered a physiotherapeutic procedure, following the institution’s standards. Usually, the time of each session was about 30 to 50 minutes, including aerobic, resistance, active, passive, and stretching exercises. The time and intensity of the physical exercise program were prescribed according to the patient’s functional level, clinical condition, and therapeutic goal.

### Bleeding frequency

It consists of simplifying the number of significant bleeds that occurred in a certain period, that is [17]:

$$Bleeding\;frequency=\frac{number\;of\;bleeds}{\Delta t}$$

It is used to quantify the frequency of bleeding in certain populations. The higher the value, the greater the occurrence of bleeding in a given time (Δ*t*). In this study, the (Δ*t*) was considered the number of days of physiotherapy intervention.

### Bleeding classification

Bleeding events were classified according to severity (Low Severity, Moderate Severity, and High Severity) and attribution to physical exercise (Highly Likely, Moderate Possibility, and Highly Unlikely) based on criteria in the literature [16]:

1. Low Severity (LS): no need for medical intervention.
2. Moderate Severity (MS): medical or nursing intervention was necessary, but it did not affect the patient’s physiotherapy intervention.
3. High Severity (HS): it resulted in the suspension of physiotherapy intervention or transfer of the patient to an environment of greater complexity.

The contribution of physical exercise to bleeding was categorized as:

1. Highly Likely (HL): extremity hematoma and cerebral haemorrhage.
2. Moderate Possibility (MP): hematuria, epistaxis, and ecchymosis.
3. Highly Unlikely (HU): hemoptysis, gastrointestinal bleeding, oral and tongue bleeding, and menstrual/vaginal bleeding.

### Data analysis

Data regarding age and hospital length of stay were expressed by the median and interquartile range. Body mass index (BMI) was expressed by the mean and standard deviation. The demographic and clinical characteristics were analysed, expressed by absolute values and percentage of the occurrence. The bleeding frequency was obtained using the formula: Bleeding frequency = Total number of bleeds/ Total number of days of physiotherapy intervention.

## Results

The sample consisted of 77 patients, 40 men (52%), and 37 women (48%), with a median age of 54 [38.7–66] years and a BMI of 25.4 ± 4.5 kg/m². The predominant type of HSCT was allogeneic (n = 41; 54%), being 30 related (39%) and 11 unrelated (15%), followed by autologous HSCT (n = 36; 46%). A total of 1341 days of physiotherapy sessions were analysed in a median hospital length of stay of 30 [23–35] days. The patients presented 1315 (98%) days of physiotherapy sessions with aerobic exercises, 1287 (96%) with resistance exercises, 36 (2.7%) with active exercises, 18 (1.3%) passive exercises, and 1341 (100%) with stretching exercises.

**Fig 1:**
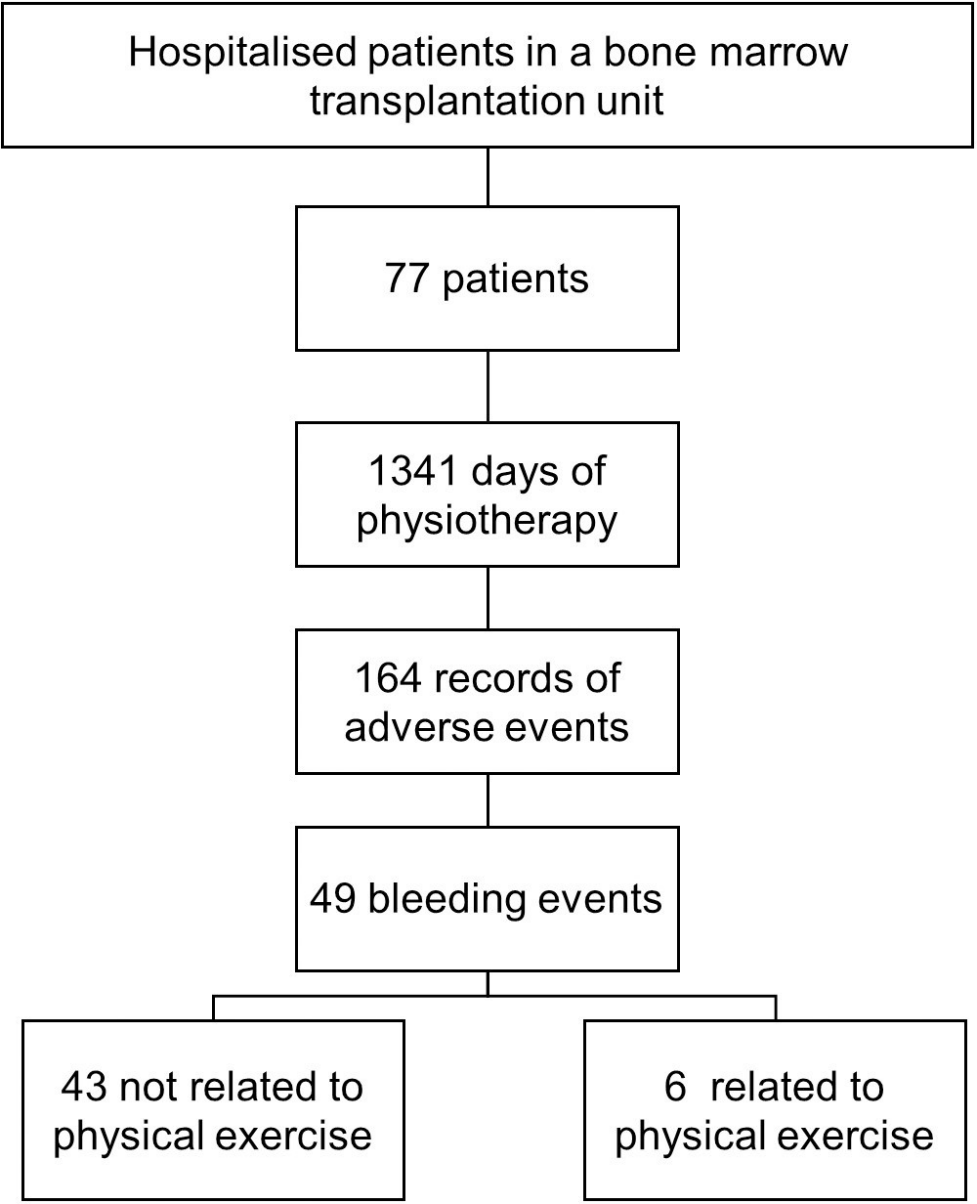
Number of patients, number of adverse events and number of bleeding-related or not to physical exercise.

A higher occurrence of platelet count was observed on admission, between 30,001–50,000 /μL (n = 60, 78%), and during the physiotherapy period between 10,001–30,000 /μL (n = 742 sessions, 55%) (Fig 1). Table 1 shows the demographic and clinical characteristics of the patients and platelet scores.

**Table 1:** Demographic and clinical characteristics of patients.

Variables	Total = 77
**Age (years)**	
18–30	13 (17%)
30–60	35 (45%)
>60	29 (38%)
**Gender**	
Male	40 (52%)
Female	37 (48%)
**BMI (Kg/m**^**2**^**)**	25.4 ± 4.5
**Ethnicity**	
White	74 (97%)
Hispanic	1 (1%)
Blacks	2 (2%)
**Primary cancer**	
Leukemia and pre-leukemic syndrome	29 (38%)
Lymphoma	24 (32%)
Multiple Myeloma	14 (19%)
Others	10 (11%)
**HSCT type**	
Allogenic	41 (54%)
Allogenic related	30 (39%)
Allogenic unrelated	11 (15%)
Autologous	36 (46%)
**Platelet count at admission (/μL)**	
<10,000	02 (03%)
10,001–20,000	04 (05%)
20,001–30,000	11 (14%)
30,001–40,000	25 (33%)
40,001–50,000	35 (45%)
**Platelet count during rehabilitation (/μL)**	
<10,000	146 (11%)
10,001–20,000	387 (29%)
20,001–30,000	355 (26%)
30,001–40,000	249 (19%)
40,001–50,000	204 (15%)

Abbreviations: BMI: body mass index; Kg: kilogram; m²: square meter; μL: microliter. Data present in mean ± standard deviation or frequency (%).

During the hospitalization, there were 164 records of adverse events; however, only 49 events were episodes of bleeding, of which, six bleeding events were related to the physiotherapy intervention, these bleeding events were present more frequently in patients with platelet levels ≤ 10,000 /μL (n = 04, 67%). These data are shown in Table 2.

**Table 2:** Bleeding events according to platelet count.

Platelet count (/μL)	Absolute Bleeding events	Absolute bleeding related to physical exercise
≤10,000	08	04 (03 Epistaxis and 01 extremity hematoma)
10,001–20,000	21	01 (Extremity hematoma)
20,001–30,000	11	-
30,001–40,000	04	01 (Hematuria)
40,001–50,000	05	-
Total	49	06

Abbreviations: μL: microliter.

Bleeding events (n = 06) were classified according to the severity and its association with physical exercise during physiotherapy intervention (Table 3). The most frequent event was epistaxis (n = 03, 50%), considered of low severity and with a moderate possibility of being related to physical exercise; followed by an extremity hematoma (n = 02, 33%), considered of moderate severity, and highly likely to be related to physical exercise in the physiotherapy intervention. In this study, there was no bleeding event considered of high severity.

**Table 3:** Bleeding characteristics, severity, and attribution to physical exercise.

	Frequency (%)
**Severity of bleeding**	
High	0
Moderate	3 (50%)
Low	3 (50%)
**Bleeding attributed to physical exercise**	
Highly likely	2 (33%)
Moderate possibility	4 (67%)
Highly unlikely	0
**Bleeding (Severity, attribution to physical exercise)**	
Epistaxis (L,MP)	3 (50%)
Hematuria (M,MP)	1 (17%)
Extremity hematoma (M,HL)	2 (33%)

Abbreviations: H: High, M: Moderate, L: Low; HL: Highly likely, MP: Moderate possibility, HU: Highly unlikely.

The bleeding frequency values for physiotherapy days are shown in Table 4. The bleeding frequency was low during physiotherapy in all ranges of thrombocytopenia, but bleeding events are more frequent in patients with platelet count ≤ 10,000 /μL.

**Table 4:** Bleeding frequency: Platelet counts (/μL) during physical therapy (number of bleeding/days of physiotherapy).

<10,000 (04/146)	0.0273
10,001–20,000 (01/387)	0.0026
20,001–30,000 (0/355)	0
30,001–40,000 (1/249)	0.0040
40,001–50,000 (0/204)	0

μL: microliter.

## Discussion

This retrospective study sample involved a greater number of male individuals (52%), aged over 30 years (83%), with a higher occurrence of related allogeneic HSCT (39%) and an average duration of hospitalization period of 32±13.27 days. Epidemiological data in Li et al. (2016) [18] found most prevalent hematologic malignancies in the male population (55.7%), and that it can be diagnosed at any age may vary from 15.3 years, as in leukemia lymphoblastic, to 77.3 years, as in chronic myelomonocytic leukemia, which may justify the mean and standard deviation of age in our study of 50.50±16.34. Data from the Worldwide Network for Blood and Marrow Transplantation (2015) [19] regarding the use of HSCT as a therapeutic intervention for hematologic disorders demonstrate increased use of the allogeneic HSCT type in Pan-American regions in recent decades. Between 1996 and 2005, 51,347 were registered, and from 2006 to 2012, 54,437 (46%) were allogeneic HSCT, corroborating the occurrence of a higher number of allogeneic HSCT in our study.

Concerning physical exercise during physiotherapy intervention, Fu et al. (2018) [16] assessed the frequency of bleeding, severity, and attribution to physical exercise in 133 patients with hematological malignance with severe thrombocytopenia (≤ 20,000/μL) during the rehabilitation period and showed 97 bleeding events, being considered of low (n = 72, 74%), medium (n = 14, 14%) and high (n = 11, 11%) severity. Regarding their relation to physical exercise, 74 (76%) were of moderate possibility, 19 (20%) were highly unlikely, and 4 (4%) highly likely to be related to physical exercise. The one event of high severity and highly attributed to physical exercise was cerebral bleeding. In this study, the bleeding rate was greater in patients with counts platelets of <10,000 and 10,000–15,000 (bleeding frequency is 0.17 and 0.37, respectively). In our study, the bleeding frequency was also more frequent in patients with thrombocytopenia <10,000 when compared to the other thrombocytopenia ranges. However, it was lower than in the study by Fu et al. (2018) (0.02 vs 0.17, respectively).

Neal et al. (2018) [20] evaluated the frequency of bleeding events in 278 cancer patients that were referred to daily rehabilitation. The bleeding was classified by the WHO bleeding scale ranging from Grade I-IV. During the rehabilitation period, 34 patients (28.6%) had at least one bleeding episode, with 12 patients having multiple bleeding episodes, totaling 56 events. Most of the bleeding events (n = 49, 87.5%) were considered to be of moderate severity rated as Grade I and II, and seven (5.8%) were considered as Grade IV. Statistical analysis showed no association between platelet count and severity of the event, including very low platelet counts (≤11,000, p = 0.106 or 11,000 to 20,000/μL p = 0.319). However, unexpectedly, there was a higher occurrence of bleeding in patients with platelet count ≥ 51,000 /μL (n = 35, 62.5%), differently from the results of our study and Fu et al. (2018) [16], which demonstrated a higher occurrence of bleeding with platelet count ≤10,000/μL but corroborates the data of a higher occurrence of low and medium severity bleeding. However, factors as the different types of cancer and the presence of patients using antiplatelet agents and anticoagulants make it more difficult to compare the studies’ results.

However, studies by Elter et al. (2009) [15] and Morishita et al. (2013) [21] did not find bleeding events in their samples. The study by Elter et al. (2009) [20] evaluated patients with hematological neoplasms who received high doses of chemotherapy and underwent submaximal aerobic exercise protocol. During the execution of the protocol, no patient with platelet count ≤10,000/μL presented bleeding and no patient with hemoglobin count ≤ 8 g/μL had severe tachycardia. Accordingly, Morishita et al (2013) [21] evaluated physical exercise in patients with allogeneic HSCT during the cytopenic period and demonstrated that no bleeding event occurred in the group that received the intervention with physical exercise. There was also no dizziness or tachycardia.

This divergence in the literature studies may be related to bleeding spontaneously occurring in patients with thrombocytopenia, in which bleeding is not related exclusively to the severity of thrombocytopenia, but the combination of multiple factors, as pointed by Ho-Tin-Noé et al. (2017) [22]. The authors suggest that the association of thrombocytopenia and the inflammatory process may induce bleeding, and not only the severity of thrombocytopenia. Also, other factors can predispose to bleeding such as age range. Toseto et al. (2013) [17] indicated a higher incidence of bleeding in older patients. This demonstrates that the cause of bleeding is multifactorial in thrombocytopenic patients and, therefore, is not only related to the severity of thrombocytopenia.

Hemorrhagic cystitis is often a serious complication of HSCT, with the potential to cause significant morbidity [23]. It occurs in up to 70% of hematopoietic stem cell transplant recipients and is associated with prolonged hospitalization [24, 25]. However, the patient who presented hematuria after exercise in the present study showed negative tests for viral or bacterial infections. In addition, showed spontaneous improvement without any clinical intervention. Multiple studies have shown that clinically significant haematuria is relatively common after exercise [26–28] and can be defined as hematuria that occurs after strenuous exercise and resolves with rest in individuals with no apparent underlying kidney or urinary tract pathology [29].

Although epistaxis occurred after physical exercise and its association with physical exercise is reported in medical records, we cannot exclude other primary causes. Spontaneous epistaxis presents several factors such as local nasal inflammation, medication, platelet and coagulation abnormalities, and hereditary hemorrhagic telangiectasia [30]. In addition, for a long time, hypertension was considered to be a major cause of spontaneous epistaxis, however, this topic appears to be more controversial in literature [31, 32]. Therefore, the present study considers epistaxis as a moderate possibility of being related to physical exercise.

Lastly, chemotherapy may induce fatigue and a severe decrease in muscle strength, especially in striated muscles which may be further aggravated by reduced physical activity [33]. In patients that did not perform physical exercise and who were receiving chemotherapy for lymphoma, there was a decrease of up to 14.6% in muscle strength [34]. In our study, we showed that the bleeding frequency is low without highly severe events. The physical exercise practice is important to enhance the gain or maintenance of muscle strength and functionality during the hospital stay in patients undergoing HSCT [35].

To summarize, the bleeding frequency was low during physiotherapy in thrombocytopenic patients undergoing HSCT in all ranges of thrombocytopenia. There was no bleeding of high severity or highly attributed to physical exercise supervised by the physiotherapist, but bleeding events of medium and low severity and medium and low attribution to physical exercise are more frequent in patients with platelet count ≤10,000 /μL.

## Conclusion

Bleeding frequency in supervised physical exercise during physiotherapy in adults with thrombocytopenia undergoing HSCT is minor and relatively rare but occurs more frequently in patients with platelet count ≤10,000 /μL. These results encourage the maintenance of physical activity in this population who is at high risk of developing immobility-related complications.
